# Automatic labeling of molecular biomarkers of immunohistochemistry images using fully convolutional networks

**DOI:** 10.1371/journal.pone.0190783

**Published:** 2018-01-19

**Authors:** Fahime Sheikhzadeh, Rabab K. Ward, Dirk van Niekerk, Martial Guillaud

**Affiliations:** 1 Department of Electrical Engineering, University of British Columbia, Vancouver, British Columbia, Canada; 2 Department of Integrated Oncology, Imaging Unit, British Columbia Cancer Research Centre, Vancouver, British Columbia, Canada; 3 Department of Pathology, British Columbia Cancer Agency, Vancouver, British Columbia, Canada; Universite de Technologie de Compiegne, FRANCE

## Abstract

This paper addresses the problem of quantifying biomarkers in multi-stained tissues based on the color and spatial information of microscopy images of the tissue. A deep learning-based method that can automatically localize and quantify the regions expressing biomarker(s) in any selected area on a whole slide image is proposed. The deep learning network, which we refer to as Whole Image (WI)-Net, is a fully convolutional network whose input is the true RGB color image of a tissue and output is a map showing the locations of each biomarker. The WI-Net relies on a different network, Nuclei (N)-Net, which is a convolutional neural network that classifies each nucleus separately according to the biomarker(s) it expresses. In this study, images of immunohistochemistry (IHC)-stained slides were collected and used. Images of nuclei (4679 RGB images) were manually labeled based on the expressing biomarkers in each nucleus (as p16 positive, Ki-67 positive, p16 and Ki-67 positive, p16 and Ki-67 negative). The labeled nuclei images were used to train the N-Net (obtaining an accuracy of 92% in a test set). The trained N-Net was then extended to WI-Net that generated a map of all biomarkers in any selected sub-image of the whole slide image acquired by the scanner (instead of classifying every nucleus image). The results of our method compare well with the manual labeling by humans (average F-score of 0.96). In addition, we carried a layer-based immunohistochemical analysis of cervical epithelium, and showed that our method can be used by pathologists to differentiate between different grades of cervical intraepithelial neoplasia by quantitatively assessing the percentage of proliferating cells in the different layers of HPV positive lesions.

## Introduction

This study addresses the automation of Immunohistochemistry (IHC) image analysis in quantitative pathology. IHC staining is a tissue-based biomarker imaging technology widely used to identify and visualize target cellular antigens specific to certain tissues. In the process, a fixed tissue sample is first sectioned and mounted on a slide, then stained with different IHC biomarkers, and finally imaged by a scanner or a charge-coupled device color camera mounted on an optical microscope. Immunohistochemistry imaging is commonly used in the detection of specific cell types or cells in specific functional states, which can ultimately reflect the physiological state and the changes in a disease process. Analysis of IHC images can determine the spatial distribution of every biomarker with respect to the tissue’s histological structures as well as the spatial relationships amongst the cells expressing each biomarker. This information could provide valuable insights to the molecular complexity of disease processes, enable new cancer screening and diagnosis tools, inform the design of new treatments, monitor treatment effectiveness, and predict the patient response to a treatment. However, the manual quantification and localization of the different subsets of cells containing a biomarker (in each IHC-stained tissue) demands a large amount of time and effort. In addition, qualitative or semi-quantitative scoring systems suffer from low reproducibility, accuracy, and sensitivity, and decreased ability to distinguish the different stains apart when they overlap spatially. Therefore, there is a need for methods that can automatically quantify IHC images. This is, however, a challenging problem due to the variations caused by the different staining and tissue processing procedures.

The techniques proposed in the literature for quantifying histopathology images are generally based on traditional image processing approaches that rely on extracting pre-defined features of nuclei or cell appearance [1,2]. Using pre-defined features in IHC image analysis however, raises the concern of low reproducibility and lack of robustness, due to variations in the nuclei and cell appearance that are caused by the different staining and tissue processing procedures used by different laboratories. Recently, several studies have shown that deep learning algorithms such as those employing convolutional neural networks [3], could be used in histopathology image analysis with great success [4–10]. For example, Jain *et al*. [4] applied a convolutional network to segment electron microscopic images into intracellular and intercellular spaces. Ciresan *et al*. [5] and Wang *et al*. [9] employed deep learning to respectively detect mitoses and metastastic breast cancer through quantitative analysis of breast histopathology images. This approach has outperformed other methods by a significant margin [5,9]. Janowczyk *et al*. [10] demonstrated that the performance of their convolutional approach in segmentation (nuclei, epithelium, tubule, and invasive ductal carcinoma), detection (lymphocyte and mitosis) and classification (lymphoma subtype) applications, was comparable or superior to existing approaches or human performance.

In this paper, we address the automation of IHC image analysis in quantitative pathology. We propose a deep learning method to detect and locate two biomarkers present in a cervical intra-epithelial lesion and quantify the percentage of proliferating cells in different layers of a p16 positive epithelium. The tissue could be a normal cervix tissue, low-grade Cervical Intraepithelial Neoplasia (CIN), or high-grade CIN. The proposed deep learning network is a fully convolutional neural network that can take an IHC image as an input, and detect and locate the different biomarkers presented in all cells of the image. This network is an extension of a convolutional neural network that is trained by using RGB images of nuclei, to classify a nucleus according to its expressed biomarker.

The benefit of using the deep learning approach (over image processing methods that rely on capturing pre-defined features of nuclei or cell appearance) is that the feature descriptors are automatically learned in the convolution kernels [11] (during the training of the network). Therefore, there is no need to find and quantify sophisticated features for every group of cells expressing a specific biomarker. We believe that by eliminating the need to find and quantify advanced features for each cell expressing a biomarker, we could develop more reproducible methods for biomarker localization in IHC images. In addition, in our proposed approach, there is no need for pre-processing the RGB images before feeding them to the network. This is in contrast to most methods in the literature, as these methods rely on complicated processing like un-mixing techniques (e.g. un-mixing multi-spectral images and color un-mixing in RGB images).

We should keep in mind that there is a drawback in employing deep neural networks. They performed very well and won grand challenges across different practical areas [11,12], and of course in the field of medical imaging [5], but the good performance of deep neural networks comes at a cost; the need for large data sets, computational complexity, and being black box predictors which can make their performances unpredictable.

In this paper, first we explain how we have collected our dataset which is composed of IHC images selected on whole slide images of cervical biopsies. Then we discuss the methods we have used to detect and localize the different biomarkers in these images. After that, we explain the experimental design, results and validation metrics we have used to evaluate our proposed approach.

## Dataset

### Patient recruitment and specimen collection

The uterine cervical biopsy specimens we used were obtained from baseline patient visits for colposcopy review at the Women’s Clinic at Vancouver General Hospital, as described in Sheikhzadeh *et al*. [13]. The samples were collected during a clinical trial over a period of 22 months (April 2013- February 2015). The study presented in this paper was part of that clinical trial. All patients had given informed consent, and the study was approved by the UBC BCCA Research Ethics Board and the Vancouver Coastal Health Research Institute (Protocols H09-03303 and H03-61235). These patients had been referred to the clinic based on prior abnormal Pap smear test results. All patients were later diagnosed as having low-grade CIN, high-grade CIN, or no abnormality (normal cervix). The uterine cervix biopsies, both normal and abnormal, were collected by trained gynecologists (DM, TE) from clinically selected sites. Biopsy specimens were immediately placed in saline after they were collected, and then transferred to the hospital pathology lab in formalin fixative. Eventually, biopsy samples were embedded in paraffin, and nine 4-μm transverse sections were cut from each of them and mounted on slides [13].

### Staining and imaging specimens

For this study, we used 4 out of the 9 slides cut from each biopsy. The other slides were used for other projects. Out of the nine slides cut from each biopsy, three slides were stained with Hematoxylin and Eosin (H&E) and reviewed by an expert pathologist (DVN) to establish disease grade as either: normal, reactive atypia, CIN1, CIN2, and CIN3. A fourth slide was double immunostained for p16/Ki-67, using the CINtec PLUS kit (Roche mtm laboratories, Mannheim, Germany) according to the manufacturer’s instruction, and then counterstained with Hematoxylin. This slide was used for immunohistochemical analysis. Our study included 69 slides in total. We randomly selected slides from biopsies with different disease grade to make sure our dataset includes all disease grades. To ensure that our analysis was not biased by our staining or tissue processing procedure, the slides were randomly assigned to one of two pathology cyto-technicians. The slides were split into seven batches (six batches of 10 slides and one batch of 9 slides). One of the two cyto-technicians stained three batches and the other technician stained four batches; one batch per week. For all the slides, one reagent lot was used and the staining was performed manually.

Each immunostained tissue sample (IHC slide) contained three labels: 1) a counterstain that labeled every nucleus (Hematoxylin [H]), 2) a chromogen (fast red) that labeled a nucleus-bound IHC biomarker (Ki-67 antigen), and 3) a chromogen (DAB) that enabled the visualization of the other IHC biomarker (p16 antigen), which is localized in the nuclei and cytoplasm [14]. The Ki-67 antigen is one of the most commonly used markers of cell proliferation, and is routinely applied to biopsies of cancerous or pre-cancerous cervical tissues [15]. The p16 protein is an important tumor suppressor protein that regulates the cell cycle, and is also commonly used as a diagnostic biomarker of cervical neoplasia [16].

We scanned each of the obtained IHC slides with a Pannoramic MIDI whole slide scanner (3DHISTECH Ltd., Budapest, Hungary) at 20X magnification. Using Pannoramic Viewer (3DHISTECH Ltd.) at 4X magnification we located the regions exhibiting the worst diagnosis on the H&E slide, and marked the same diagnostic areas on the corresponding CINtec stained slide. Then, the marked areas were exported to RGB images in TIFF format (with the total magnification of 80X and the resolution of 96 dpi). A total of 69 images, varying in size from 1024×1024 up to 17408×17408 pixels, were used in our analysis and validation. For simplicity, we will refer to these images as IHC images in the rest of this paper.

## Biomarker labeling method

**Fig 1:**
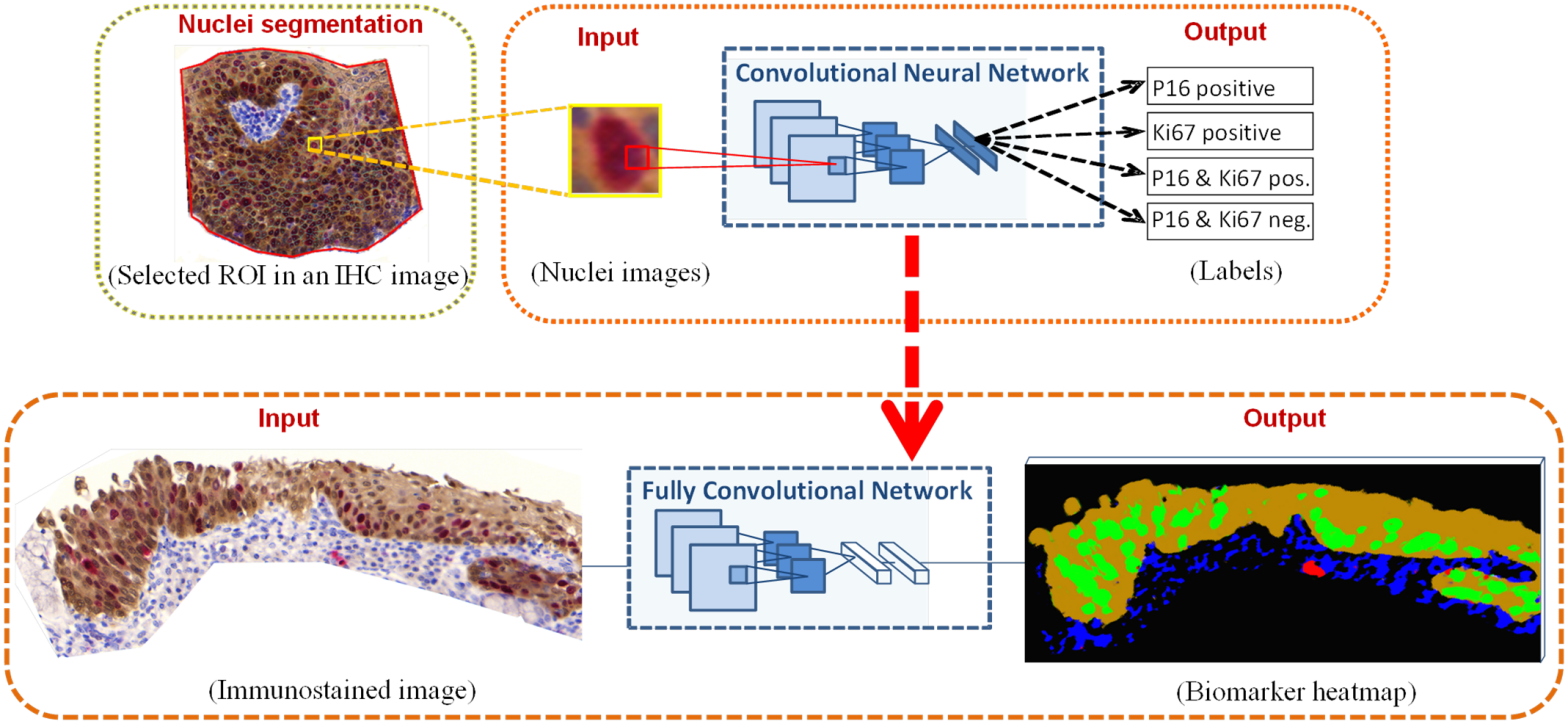
A two-step approach to biomarker labeling in IHC images. Step one (top): N-Net was trained with nucleus images segmented from IHC images in the training set, where each image had one nucleus only. This network learned to classify each nucleus image according to the expressed biomarker(s) in its respective nucleus. Step two (bottom): the trained classifier (N-Net) was extended to WI-Net. This end-to-end, pixel-to-pixel network localized biomarkers of the IHC images. The input to the WI-Net is an IHC image and the output is a heat-map of the two biomarker expression.

The biomarker labeling, outlined in Fig 1 was done in two main steps: i) training a convolutional neural network which we will refer to as Nuclei(N)-Net to label the expressed biomarkers in one nucleus, and ii) transforming the N-Net to a fully convolutional network to locate all cells expressing each of the different biomarkers in an IHC image. We will refer to this network as Whole Image (WI)-Net.

In the first step, the nuclei in the IHC images were segmented. The resulting nuclei images were labeled and then fed as inputs to the N-Net. We would like to clarify that in our segmentation, we did not aim to delineate the exact border of the nucleus or the cytoplasm (which was not necessary for our application) and therefore, each nucleus image was represented by a square image consisting of the nucleus and its surrounding cytoplasm. The outputs of the N-Net were the labels that identified the expressed biomarker in the nucleus/cytoplasm of each input image (Fig 1, top). In the second step, a whole IHC image of arbitrary size was fed into the WI-Net, and for each pixel the probability of each biomarker being expressed was calculated. These probability values were ultimately used to produce a color heat-map of the different regions expressing different biomarkers in the IHC image (Fig 1, bottom).

### Nuclei classification using N-Net

**Fig 2:**
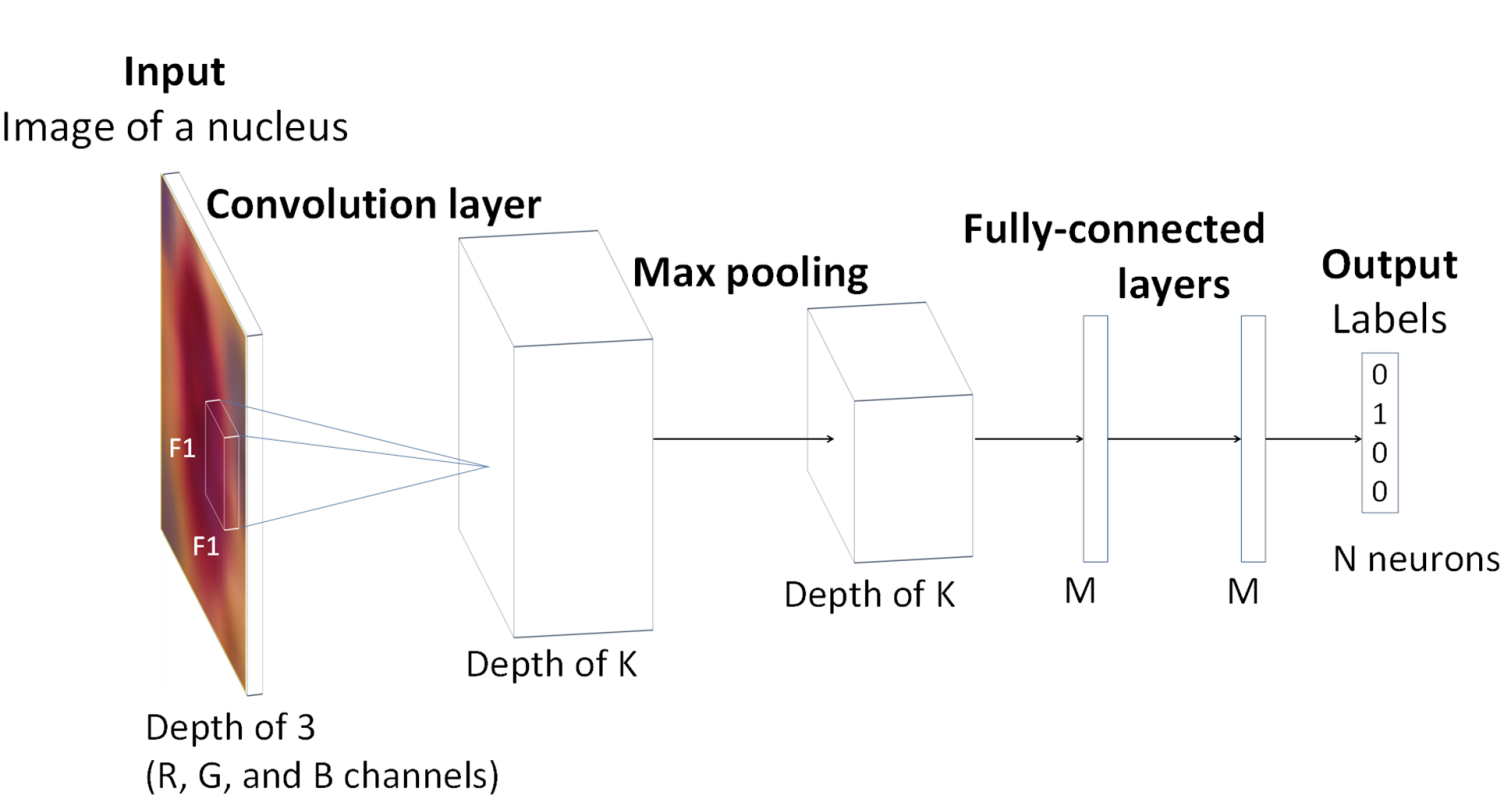
Schematic representation of the architecture of the N-Net. N-Net consists of one convolution layer, followed by a max-pooling layer and two fully connected layers. The last layer is the output layer. This network takes an RGB image of a nucleus as input and generates a label as the output.

The N-Net was the classifier we built for labeling the expressed biomarkers in nuclei images. Fig 2 shows the schematic representation of the N-Net configuration, which is a convolutional neural network [3] consisting of five layers. The input to the first layer is an RGB image containing one nucleus only. The first layer is a convolution layer that convolves the input image with K filters, each having a kernel size of F1× F1×3. These kernel matrices are learned by the N-Net. The second layer is a max-pooling layer with a kernel size of F2 by F2, which is a sub-sampling layer that reduces the size of the image. The third and forth layers are fully connected layers, each containing M neurons. The output layer consists of N neurons, each with a Gaussian function. This layer generates labels, showing the probability of each biomarker being present in the image.

### Biomarker localization in whole slide images using WI-Net

**Fig 3:**
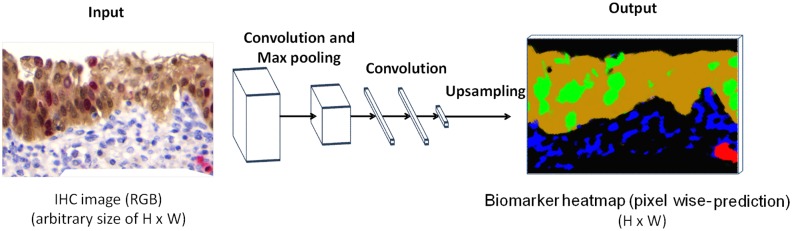
Schematic representation of the architecture of the WI-Net. The first two layers of WI-Net (convolution and max-pooling layers) are the same as the first two layers of N-Net illustrated in Fig 2. The last three layers of WI-Net are convolution layers. The input to the WI-Net is a whole IHC image, and the output is a heat-map of the present biomarker(s) in the IHC image.

In the previous section, we explained how N-Net was trained as a classifier for images, each containing one nucleus. In order to employ this classifier for localizing biomarkers on a whole IHC image (containing thousands of nuclei), different approaches could be used. For example, a sliding window (whose size is equal to the N-Net input size) could be employed on each pixel of the IHC image. Taking the window region as the input of the N-Net and obtaining the N-Net output, could generate a label for each pixel in the IHC image. The main disadvantage of this approach is that it could get computationally intensive especially for large input images. Therefore, we used a more computationally optimized approach in this study, and took advantage of the nature of convolution which could be considered as a sliding window [17]. By transforming the trained N-Net to WI-Net which is a fully convolutional network, a classification map of the IHC image is obtained (rather than generating a single classification of a nucleus image by the N-Net). This transformation is shown in Fig 3. The fully connected layers of the N-Net were converted to convolutional layers [17]; this was done for the purpose of modifying these layers to make them independent of the spatial size.

## Experiments and results

### Biomarker localization

#### Preparing nuclei images for training N-Net

**Fig 4:**
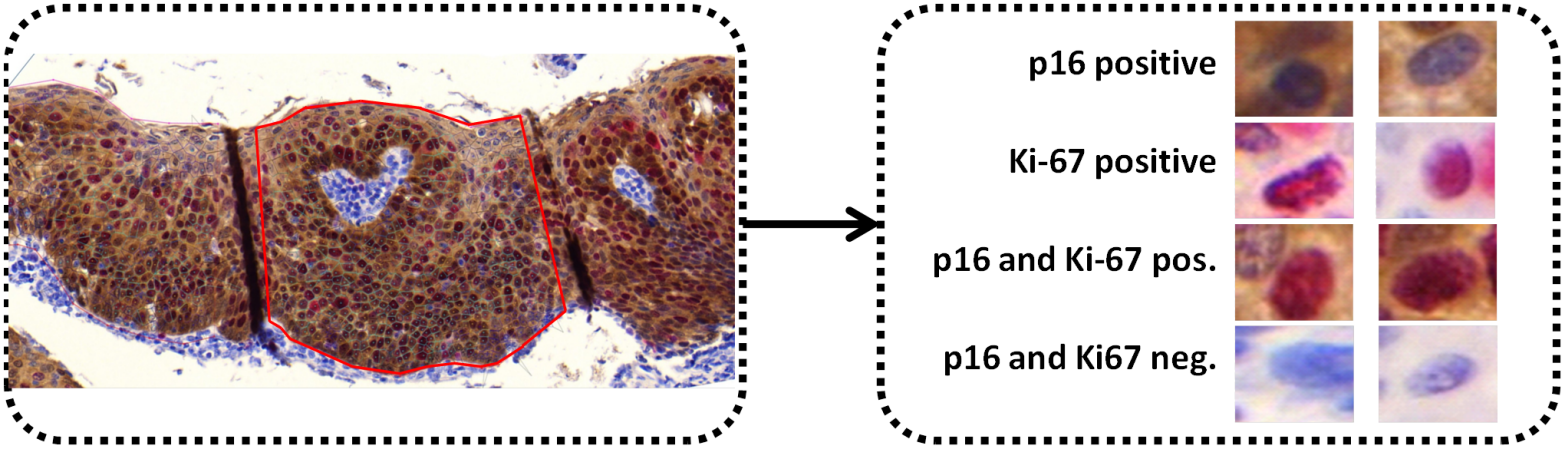
Sample of nuclei images obtained from an IHC image. ROI (red enclosure) selected in an IHC image, within which the nuclei were segmented in order to obtain nuclei images for training N-Net (left). Nuclei images expressing different proteins (right); p16 positive, Ki-67 positive, p16 and Ki-67 positive, and p16 and Ki-67 negative.

To train N-Net, we must first prepare nuclei images from the whole slide IHC images. To do so, we first selected different regions of interest (ROI) delineating the basal membrane and the superficial membrane in six whole slide images and then coarsely segmented the nuclei in each ROI. Fig 4 shows the ROI selection, the nuclei images and the label corresponding to each of four different protein expressions (p16 positive, Ki-67 positive, p16 and Ki-67 positive, and p16 and Ki-67 negative).

To select the ROIs we used our in-house image analysis software, Getafics [18], and manually delineated the basal membrane and the superficial membrane within each image. Then, we detected the nuclei and their centers of gravity within each ROI at magnification of 20X. This procedure was semi-automated, requiring only minor manual adjustments (described in [19]).

Digital RGB images, each containing one nucleus, were rescaled to 32 by 32 pixel images and stored in a gallery. We then manually labeled the nuclei into four groups: i) p16 positive, ii) Ki-67 positive, iii) both Ki-67 and p16 positive, and iv) both p16 and Ki-67 negative. These nuclei images along with their group number were considered as the ground truth and used for training the N-Net.

We had a total of 4679 labeled nuclei RGB images obtained within the ROIs from the six IHC images. The IHC images were acquired from six cases: two CIN3, one CIN2, two CIN1 and one negative (normal cervical tissue) cases. Of these 4679 nuclei images, 156 nuclei images belonging to one of the six IHC images were used for testing the performance of N-Net, and the remaining 4523 nuclei images obtained from the other five IHC images were used for training. Table 1 illustrates the distribution of the nuclei in the training and test sets of the different groups.

**Table 1:** Distribution of nuclei in the training and test sets.

	Number of nuclei in the different groups				
	p16 positive	Ki-67 positive	Ki-67 & p16 positive	p16 and Ki-67 negative	Total
**Train Set**	918	866	1572	1167	4523
**Test Set**	62	25	52	17	156

#### Training and testing N-Net

We used the following network configuration. As mentioned above, the first layer of N-Net was a convolutional layer containing 96 filters with a kernel size of 11×11×3 and stride of 4 through which each filter was slid. The second layer was a max-pooling layer with a kernel size of 3×3, and stride of 2. In order to define the kernel sizes, we used a validation set consisting of 161 nuclei images and trained the N-Net with different kernel sizes. The kernel size corresponding to the lower error rate was selected. The third and forth layers were fully connected layers, each containing 2048 neurons. The output layer consisted of four neurons. The N-Net was implemented by using the open source, deep learning framework Caffe [20], and a system with an Intel I5-6600 3.3GHZ Quad core CPU and a GTX750TI GP.

For training, we used the back-propagation algorithm whereby 4523 labeled nuclei images were the inputs to the N-Net. The Softmax function was used in the output layer of the N-Net. We considered four labels (representing the four groups) as the output of the N-Net. The trained network learned the features corresponding to the presence of following biomarkers in each nucleus: i) H and p16 (label: 0 0 0 1), ii) H and Ki-67 (label: 0 0 1 0), iii) H and both Ki-67 and p16 (label: 0 1 0 0), iv) H only (absence of Ki-67 and p16) (label: 1 0 0 0). Training was stopped after 8000 iterations (with an error rate of 0.04 on the training set). Following the training, we performed the classification on the test set using N-Net. Tables 2 and 3 show the confusion matrix of the results of the training and test sets respectively. According to the confusion matrix, the classification error rate for N-Net was 0.07 on the test set.

**Table 2:** Confusion matrix of the classification results on the training set using N-Net.

Training Set					
	**p16 pos. (Predicted)**	**Ki-67 pos. (Predicted)**	**Ki-67 & p16 pos. (Predicted)**	**p16 & Ki-67 neg. (Predicted)**	**Accuracy**
**p16 pos. (Actual)**	869	0	45	4	94.6%
**Ki-67 pos. (Actual)**	0	835	30	1	96.4%
**Ki-67 & p16 pos. (Actual)**	79	10	1483	0	94.3%
**p16 & Ki-67 neg. (Actual)**	1	6	0	1160	99.4%
**Overall Accuracy**					96.1%

**Table 3:** Confusion matrix of the classification results on the test set using N-Net.

Test Set					
	**p16 pos. (Predicted)**	**Ki-67 pos. (Predicted)**	**Ki-67 & p16 pos. (Predicted)**	**p16 & Ki-67 neg. (Predicted)**	**Accuracy**
**p16 pos. (Actual)**	59	0	1	2	95.1%
**Ki-67 pos. (Actual)**	0	25	0	0	100%
**Ki-67 & p16 pos. (Actual)**	8	0	44	0	84.6%
**p16 & Ki-67 neg. (Actual)**	0	0	0	17	100%
**Overall Accuracy**					92.9%

To estimate the accuracy of N-Net classification, we also performed a 10-fold cross-validation analysis. We split the training set (4523 nuclei images) in to 10 subsets (nine sets of 452 nuclei and one set of 455 nuclei). The subsets were approximately balanced in different labels. We used the same network architecture and training parameters as explained above and trained the network 10 different times. At each training round, we trained the N-Net by using 9 subsets and then test the network performance on the left out subset. In 10 training rounds, we obtained the average accuracy of 0.94 (SD = 0.01) on classifying the test subsets. The obtained accuracy is consistent with the results obtained from training the N-Net with the train set of 4523 nuclei images and testing its performance on an independent set of 156 nuclei images. The results suggest that the performance of N-Net is robust to the way we split our dataset into training and test data.

#### Labeling biomarkers in IHC images using WI-Net

**Fig 5:**
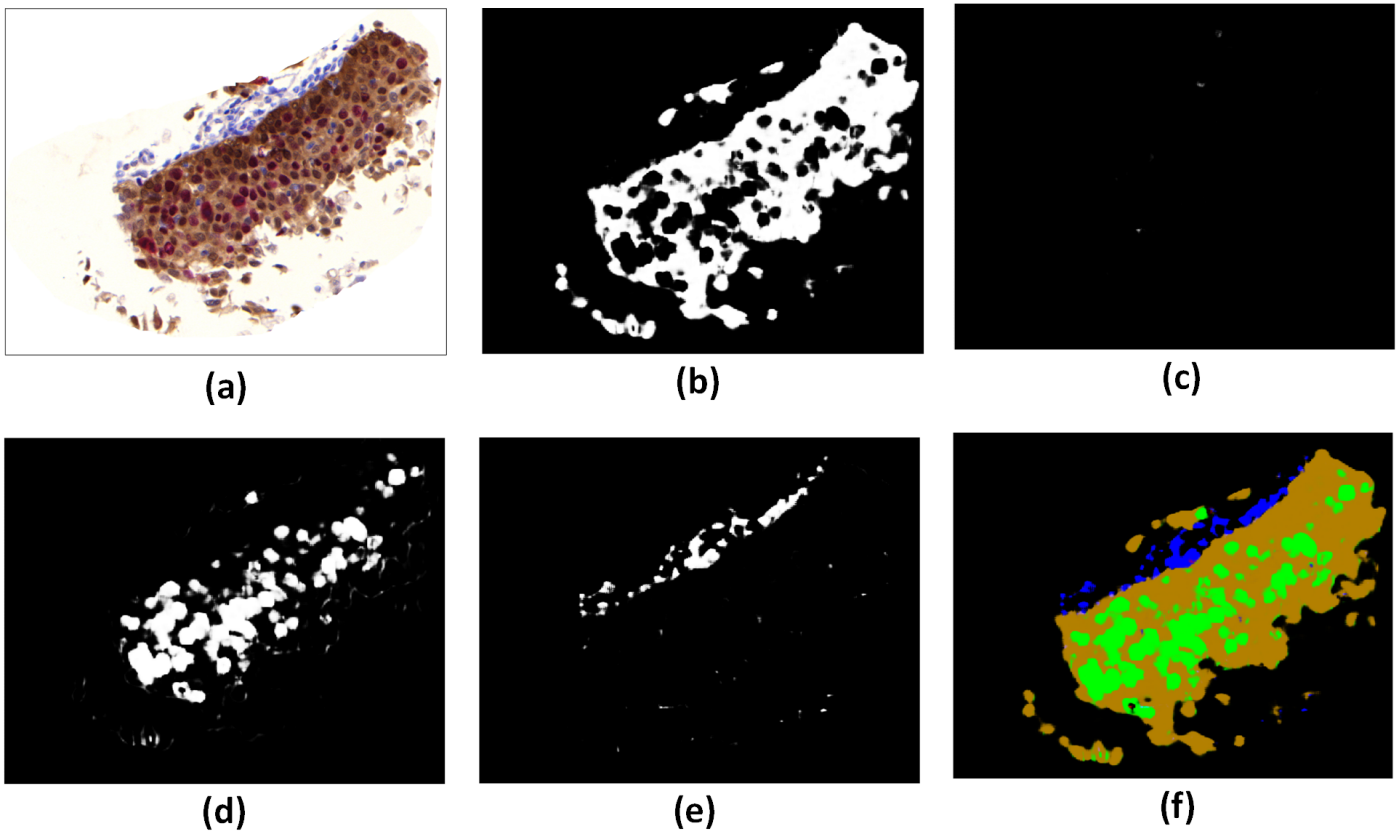
Heat-maps resulting from biomarker labeling on an example IHC image (a) using WI-Net. The IHC image (size 2048×2048 pixels) belonging to a whole slide image obtained from the scanner was fed to the WI-Net (a). The outputs of the WI-Net were the heat-maps (size 2048 × 2048 pixels) marking p16 positive regions (b), Ki-67 positive regions (c), p16 and Ki-67 positive regions (d), p16 and Ki-67 negative regions (e). The four heat-maps were combined to produce an overall biomarker heat-map (f).

After training the N-Net in labeling the expressed biomarker(s) in nuclei image, we transformed the N-Net into WI-Net for analyzing the whole IHC image. The WI-Net produced a classification map that covers an input image of arbitrary size (*i*.*e*. the produced classification map is not the result of a single classification). We transformed the N-Net to WI-Net based on the method presented by Shelhamer *et al* [17]. The kernel sizes for WI-Net are the same as N-Net. The only difference is the last three layers, which are fully connected layer in N-Net and we transfer them to convolutional layers (with the kernel sizes of 4, 1, and 1 respectively) in WI-Net. Next, we fed each IHC image as an input to the WI-Net, and obtained a heat-map for each of its biomarker. In total four heat-maps were produced for each IHC image, each illustrating the regions in the IHC image that are: i) p16 positive, ii) Ki-67 positive, iii) both p16 and Ki-67 positive, and iv) both p16 and Ki-67 negative (only expressing H). Fig 5 shows an example of an IHC image (Fig 5a) and its corresponding heat-map obtained as the output from the WI-Net (Fig 5f).

Visual comparison of the original IHC image and the labeling results from WI-Net (Fig 5a and 5f) shows that the WI-Net performance in localizing p16 and Ki-67 in the dual-stained tissue was qualitatively acceptable. In the following section we provide more validation for our method.

### Validation

**Fig 6:**
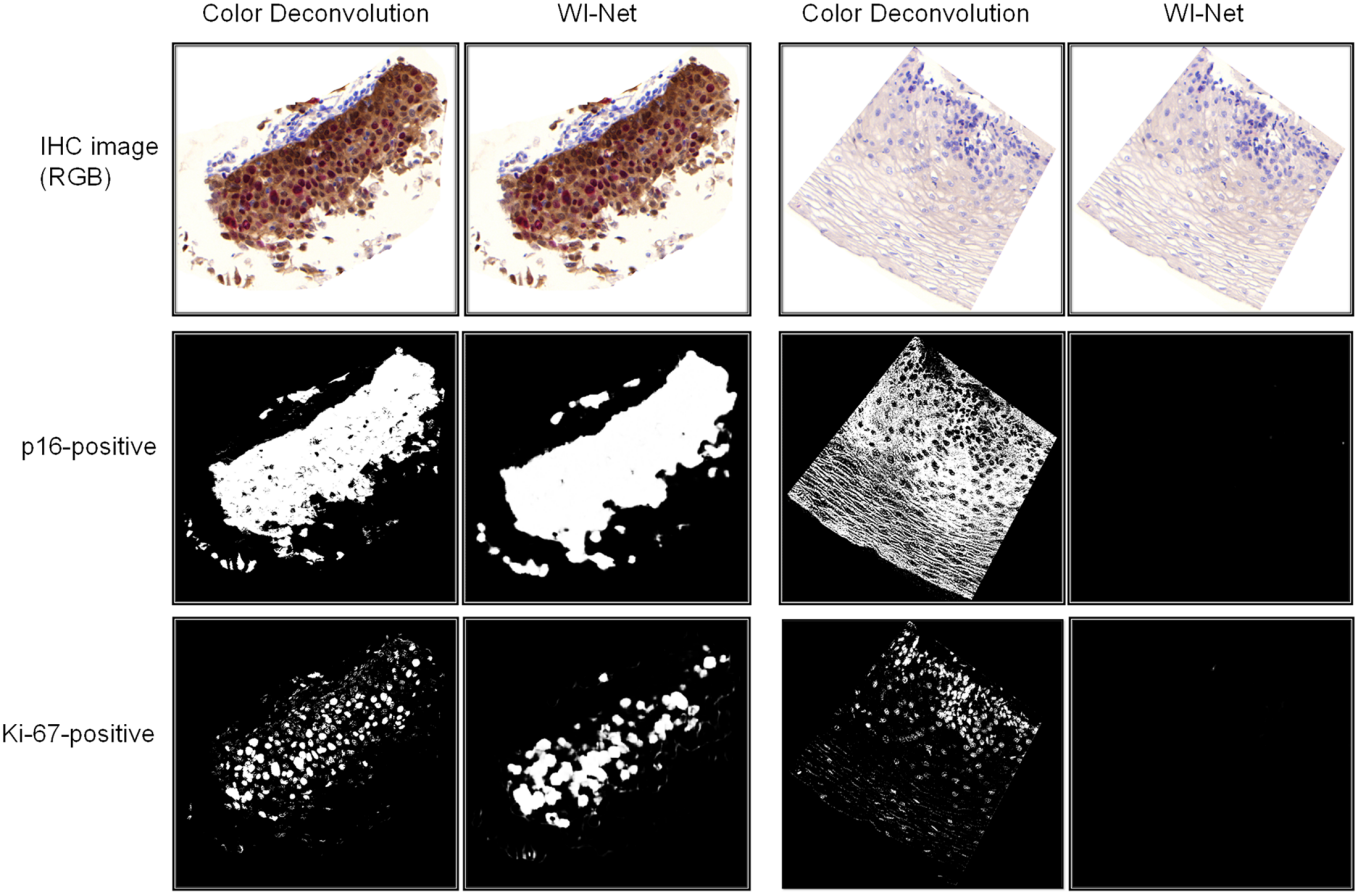
Comparison between color deconvolution approach and WI-Net approach for locating p16 and Ki-67 positive pixels in two IHC images. The IHC image on the left two columns corresponds to a high-grade cervical lesion. The IHC image on the right two columns corresponds to a normal cervical epithelium. The first row shows the RGB images. The second row shows the regions marked as p16 positive by the two methods. The third row shows the regions marked as Ki-67 positive by the two methods.

To evaluate the performance of our method, we compared the results obtained from WI-Net with those obtained from a color deconvolution approach [21]. We employed the color deconvolution presented by Ruifrok et al. (using Fiji implementation [22]) which is one of the most widely used color un-mixing approaches in digital pathology. Fig 6 shows the results of locating p16 and Ki-67 on two IHC images acquired from two dual-stained tissue specimens (one CIN3, and one normal tissue) by using color deconvolution and WI-Net.

In locating p16 and Ki-67 in the CIN3 tissue (where both p16 and Ki-67 were present), we observed that the color deconvolution method performed very well. A closer look at the results reveals that the presence of p16 in the CIN3 tissue was slightly under-estimated by the color deconvolution method (in central areas of the tissue), while it was slightly over-estimated by our WI-Net. On the other hand, some regions that are Ki-67 negative are marked as Ki-67 positive by the color deconvolution method. It should be noted that the knowledge of the exact border of a nucleus or a cell in immunostain quantification is not critical, and the main concern is to detect the presence of a biomarker in a cell. Hence, in general, we could say that the performances of color deconvolution and WI-Net in localizing p16 and Ki-67 in CIN3 tissue were comparable.

In the normal tissue (where none of p16 or Ki-67 were present), we observed that our WI-Net method did not classify any pixel as p16 positive or Ki-67 positive. The color deconvolution method however had mistakenly marked some regions on the tissue as being p16 or Ki-67-positive. The lack of robustness in the color deconvolution method over different tissue samples with different disease stages, could be due to difficulty in finding the optimal parameter/threshold setting. It is possible that with a set of parameters the color deconvolution would perform better in high-grade tissues, but would fail in low-grade tissues.

In addition, we performed two separate analyses in order to evaluate the efficacy of our proposed biomarker labeling method. First, we compared the performance of our fully automatic algorithm to human performance. Second, we investigated the utility of our immunohistochemical analysis to quantify the proliferation characteristics of the epithelium to aid with delineating between different pathology grades.

#### Comparison with manual biomarker labeling

We manually labeled the cells expressing different biomarkers in IHC images, and then used the labeled cells as the ground truth. Manually obtaining a biomarker heat-map for each IHC image is not easy; it is extremely laborious, requiring manual delineation of the membrane around each nucleus and cell and labeling each pixel in an IHC image. However, the knowledge of the exact border of a nucleus or a cell in immunostain quantification is not critical, and the main concern is to detect the presence of a biomarker in a cell. Hence, we only marked the center of the nucleus in each cell and assigned a label to the center based on the expressing protein(s). To obtain the best possible manual labeling (in the sense that it could be regarded with confidence as the ground truth for our comparisons), we asked two experienced cyto-technicians to assign these labels. Basically, the cyto-technicians worked together and reached to an agreement on the protein expression for each cell. We considered the label assigned to the center of a nucleus as the ground truth of the expressed protein(s) in its corresponding cell. We compared this ground truth to the biomarker heat-map generated by the outlined proposed method. For each marker, we defined the following metrics:

- Number of correctly labeled nuclei (true positive): this is the number of nuclei centers in the ground truth which are marked positive for that biomarker and are classified as positive for the biomarker heat-map.
- Number of missed nuclei (false negative): this is the number of nuclei centers in the ground truth which are marked positive for that biomarker and are classified as negative in the biomarker heat-map.
- Number of falsely labeled nuclei (false positive): this is the number of nuclei centers in the ground truth which are not marked positive for that biomarker but are classified as positive for the biomarker heat-map.

Table 4 shows the validation metrics for the WI-Net performance compared to the human performance (the ground truth). From the above metrics, we calculate the precision, recall, and the F-score [23]. We selected two IHC images from our data set and using the proposed method, we generated their biomarker heat-maps. These images were never seen before by the network; they were not used (neither as nucleusimages or whole images) for training or testing the N-Net or WI-Net.

**Table 4:** WI-Net performance evaluation of two IHC images taken from two WSIs: Based on biomarker heat-maps generated by WI-Net. The ground truth is taken as the labels obtained manually by humans.

	IHC image 1 (high-grade CIN)			IHC image 2 (normal epithelium)		
**Image size**	2048×2048 pixels			3072×3072 pixels		
**Process time**	166 sec			328 sec		
**Biomarker**	p16 pos.	Ki-67 pos.	p16 & Ki-67 neg.	p16 pos.	Ki-67 pos.	p16 & Ki-67 neg.
**True positive**	271	157	0	0	18	253
**False positive**	1	12	0	0	2	28
**False negative**	0	0	0	0	0	2
**Recall**	0.996	0.929	-	-	0.900	0.900
**Precision**	1	1	-	-	1	0.992
**F-score**	0.998	0.963	-	-	0.947	0.943

#### Immunostain analysis in differentiating pathology grades

The true validation of any algorithm in medical imaging requires the evaluation of how it performs in accordance with biological behavior/ outcome. Currently, p16 staining is used to differentiate between low-grade or precancerous tissues where morphological interpretation is not sufficient. The information obtained from this staining could also be used to better manage high-grade lesions [24]. In addition, there is a relationship between how proliferated cells are distributed in the epithelial layer of the tissue and the grade of neoplasia [14,25]. In higher grade CINs, proliferation would be seen anywhere from basal membrane to the surface of the epithelium, while in lower grades it is commonly seen in the basal layer and rarely in upper layers of epithelium. Furthermore, studies have suggested that the coexpression of p16 and Ki-67 may help to differentiate high-grade dysplasia from other benign lesions [14–16,25].

In this section, we investigate the performance of our biomarker labeling algorithm in quantifying the proliferation characteristics of the epithelium for discriminating between different CIN grades. As previously described, three H&E-stained slides and one CINtec-stained slide were cut from each biopsy and used in this study. The study pathologist (DVN) determined the CIN grade for each biopsy and selected the diagnostic areas in the H&E-stained slide; two trained pathology technicians drew ROIs on the image of CINtec-stained slide, marking those areas. More than one region could be indicative of worst diagnosis, therefore the technicians drew as many ROIs of any size as needed on each image (the ROIs do not overlap). The coordinates of these ROIs were saved and used in validation.

**Fig 7:**
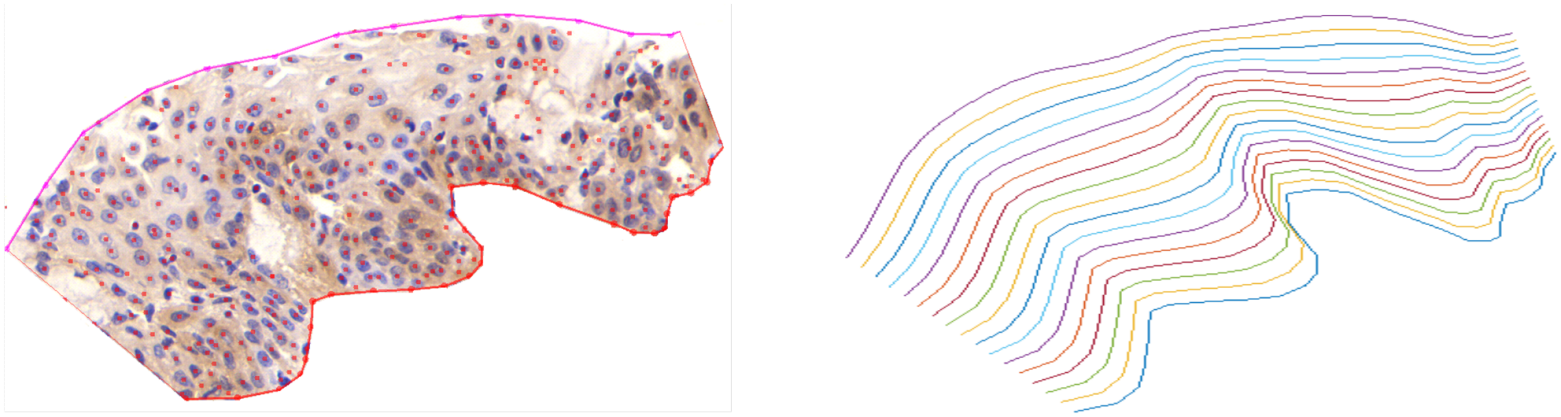
The ROI selected on an IHC image (left), and evenly distributed layers in the ROI parallel to the basal layer (right).

The proposed biomarker labeling algorithm was performed on all CINtec-stained images, and heat-maps of biomarkers (Ki-67 and P16, in this study) were obtained for each image. As mentioned before, the input to the algorithm were raw RGB images of arbitrary size selected from immunostained WSIs obtained from scanner. A total of 61 IHC images, varying in size from 2048×1024 up to 17408×17408 pixels, were analyzed. The entire process was fully automated and fast (approximately 30 seconds for analyzing an image of size 1024×1024 pixels). For each case, the coordinates of the ROIs drawn by technicians were used to obtain the equivalent ROIs on each heat-map. Each ROI was then divided into *n* layers, all of which were parallel to the basal layer and evenly distributed from the basal membrane to the top of the epithelium (Fig 7).

**Fig 8:**
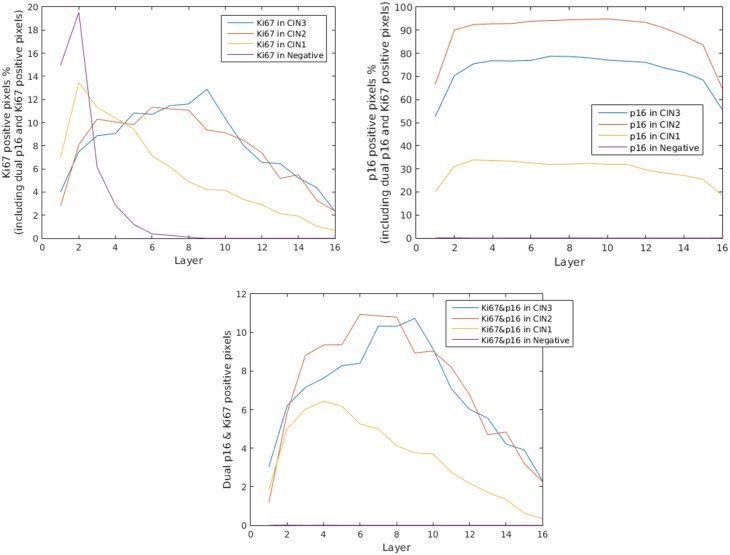
The average percentage of pixels classified as Ki-67 positive (top left), p16 positive (top right), and both Ki-67 and p16 positive (bottom) in different layers of images of cervical epithelium as assessed for each pathology grade. Layer 1 is the first layer (i.e. basal layer) and layer 16 is the superficial layer.

Using these layers, we obtained the percentage of positive pixels for each stain in each layer. The graphs in Fig 8 show the average percentage of positive pixels in different layers of epithelium. The results shown in Fig 8 were obtained by analyzing a total of 94 ROIs marked on 61 IHC images. Out of these 61 slides, 16 were CIN3 (31 ROIs), 13 were CIN2 (19 ROIs), 15 were CIN1 (24 ROIs), and 17 were negative (20 ROIs). Testing was done to determine how many layers n to use. For different number of layers *n* from 10 to 20, no significant variation was observed in the results, therefore we chose to set *n* to 16.

As shown in Fig 8, the relative proliferation pattern obtained from the results is in good accordance with the patterns reported in the literature [25]. In normal epithelia, proliferation mainly occurred in first layers (closer to basal membrane) and dropped rapidly in the upper layers. The patterns observed in CIN1, CIN2 and CIN3 epithelia were similar to each other in the first layers (up to one fourth of the thickness of the epithelium). Interestingly, the proliferation rate was notably higher in the upper layers of CIN2 and CIN3 epithelia comparing to CIN1 epithelia.

We observed negligible p16 positive pixels in the layers of normal cervical tissue images. For all the layers, the average percentage of p16 positive pixels in low-grade CIN was noticeably lower than the average of positive pixels in high-grade CIN (Fig 8). The results show that the layer-based co-expression of p16 and Ki-67 as a metric has the potential to be employed for differentiating between normal, low-grade, and high-grade CIN. The dual positive pixels were not seen in any layer of normal tissue. Although the percentage of dual positive pixels were similar in the two first layers of low-grade and high-grade CINs, this percentage in low-grade CIN dropped to half of the percentage in high-grade CINs in upper layers (starting from the half of the thickness of the epithelium).

## Discussion

Immunohistochemical analysis is commonly employed for detecting dysplastic cells in pre-neoplastic lesions and for localizing the expression of one or more proteins in a tissue. In IHC images, it is important to localize the cells that express each of the different labels and visualize the different biomarkers. In this paper, we propose a deep learning-based algorithm for the immunohistochemical analysis of IHC images of arbitrary sizes. The IHC images used in this study were images of selected regions in whole slide images, acquired by whole slide scanners. However, it is worth noting that, if required in an application, the proposed algorithm can be easily employed to process the whole slide image as well. Considering that the input size is not fixed for this algorithm, it can process images of arbitrary sizes using the proper hardware. The results from our method are in accordance with the expert human observers’ classification and compare well with the labeling performed manually by experienced cyto-technicians.

We also compared the performance of our WI-Net method for localizing p16 and Ki-67 on dual-stained tissues with the performance of the color deconvolution method presented by Ruifrok *et al*. [21]. We observed in high-grade tissues, their performance were comparable, but in low-grade tissues, where p16 and Ki-67 were missing, our method performed superior to the color deconvolution method (Fig 6). This could be a result of manual parameter/threshold setting in color deconvolution method. One set of parameters may result in very good labeling in high-grade tissue, while failing in labeling biomarkers in low-grade tissues. In general, adjusting threshold/parameters in such un-mixing techniques could be very subjective. In the approach presented in this paper, there is no need to change any parameters manually. Even for employing our method on unseen data from another institution, there is no need to change the architecture of the neural network or the training procedure. The network could tune its parameters during the training with a small dataset from the new institution. The effort needed to fine-tuning a pre-trained network for each institute is much less than the effort than needed for training the network from scratch.

One main challenge that our method tackled was to automatically quantify the proliferation characteristics of the epithelium on dual-stained tissues. Ki-67 and p16 presentation can look different in each slide or even in different regions in the same slide. It is very difficult for human observers to identify the cells that are Ki-67 positive in a dual-stained tissue, when the cells are also p16 positive. To human observers, the color appearance of these cells could be very similar to the color appearance of the cells that are p16 positive and Ki-67 negative. Even the expert cyto-technicians that have prepared our dataset for training and testing our N-Net had difficulties to agree on the expression of Ki-67 protein in p16 positive cells. Therefore, we decided to use nuclei images as basic elements for our training. We trained the network with many nuclei images that were p16 and Ki-67 positive or negative, so that the network got exposed to real examples of a p16 or ki67 positive cell appearance, and learned the features that were related to these protein expressions.

In this study, the color and spatial information of each pixel in an IHC image (which is an RGB image) were used to identify the different biomarkers and localize the cells expressing each biomarker (or a combination of them) in one integrated step. In this approach, the color information was not disregarded in training the WI-Net, as color plays an important role in immunohistochemical analysis. There is no need to perform any pre-processing on the color RGB images. The color IHC images were fed directly to the network. In contrast, most methods in the literature include complicated processing like an un-mixing technique (e.g. un-mixing in multi-spectral images and color un-mixing in RGB images). For example, in the study by Chen *et al*. [6] (in which convolutional neural networks was employed in immunohistochemical analysis), a sparse color un-mixing algorithm to un-mix the RGB image into different channels representing different markers was used. Then, a convolutional neural network was used as a cell detection tool in one channel.

The real ground truth is the biological behavior or lesions outcome, and one can only validate the performance of the IHC biomarker quantification by correlating the results with these biological behavior. Therefore, in addition to visual evaluation of the classification results and comparison against manually-performed labeling, and visual comparison between our method and a color deconvolution method, we used our method to automatically obtain different immunostain expression patterns in cervical epithelia with different pathology grades. We have shown that there is a correlation between these patterns and the pathology grades (Fig 8).

Based on these results, it can be concluded that the proposed method has the utility to be used for discriminating between different grades. More importantly, it may aid the pathologist to distinguish between CIN1 and CIN2 in difficult cases. Since diagnostic of CIN1 or CIN2 may eventually result in different therapeutic management, any novel method (including imaging biomarker) that can help pathological decision should be carefully assessed. We should also consider the fact that deep learning-based algorithms are black box predictors which could make them especially risky in a clinical setting. Now that such algorithms and tools are being developed, extensive studies over multiple institutions should be done to examine the reliability of these algorithms.

## Conclusion

We trained an end-to-end network for pixel-wise prediction of molecular biomarkers in immunostained whole slide images. The results demonstrate that the proposed automatic labeling method compare well (average F-score of 0.96) with the labeling (p16 positive, Ki-67 positive, p16 and Ki-67 positive, and p16 and Ki-67 negative) performed manually by expert cyto-technicians. The advantage of this method is that it eliminates the need for finding and quantifying sophisticated features of those cells that express a specific biomarker. Therefore, this approach is potentially more reproducible than methods that rely on extracting pre-defined or hand-crafted features of nuclei or cell appearance. In addition, we have shown that it provides an accurate assessment of epithelial proliferation characteristics that could be helpful in delineating disease grades. It should be noted that this approach could be easily applied to different tissue types and different stains combinations and other research questions in modern pathology.

## Supporting information

S1 Fig Expression of p16 and Ki67 proteins per layers for Cervical Intraepithelial Neoplasia 3 (CIN3) lesions. Expression of p16 and Ki-67 per layers in CIN3 lesions. The box plot of the percentage of pixels classified as Ki-67-positive (top left), p16-positive (top right), and both Ki-67 and p16-positive (bottom) in the different layers of CIN3 lesions. Layer 1 corresponds to the first layer (i.e. basal layer) and layer 16 corresponds to the most superficial layer. (*Central point* median; *box* first and third quartiles; *whiskers* most extreme data points not considered outliers, + outliers).
(TIF)

S2 Fig Expression of p16 and Ki-67 proteins per layers for CIN2 lesions. Expression of p16 and Ki-67 per layers in CIN2 lesions. The box plot of the percentage of pixels classified as Ki-67-positive (top left), p16-positive (top right), and both Ki-67 and p16-positive (bottom) in the different layers of CIN2 lesions. Layer 1 corresponds to the first layer (i.e. basal layer) and layer 16 corresponds to the most superficial layer. (*Central point* median; *box* first and third quartiles; *whiskers* most extreme data points not considered outliers, + outliers).
(TIF)

S3 Fig Expression of p16 and Ki-67 proteins per layers for CIN1 lesions. Expression of p16 and Ki-67 per layers in CIN1 lesions. The box plot of the percentage of pixels classified as Ki-67-positive (top left), p16-positive (top right), and both Ki-67 and p16-positive (bottom) in the different layers of CIN1 lesions. Layer 1 corresponds to the first layer (i.e. basal layer) and layer 16 corresponds to the most superficial layer. (*Central point* median; *box* first and third quartiles; *whiskers* most extreme data points not considered outliers, + outliers).
(TIF)

S4 Fig Expression of p16 and Ki-67 proteins per layers for normal squamous cervical epithelia. Expression of p16 and Ki-67 per layers in normal squamous cervical epithelia. The box plot of the percentage of pixels classified as Ki-67-positive (top left), p16-positive (top right), and both Ki-67 and p16-positive (bottom) in the different layers of normal squamous cervical epithelia. Layer 1 corresponds to the first layer (i.e. basal layer) and layer 16 corresponds to the most superficial layer. (*Central point* median; *box* first and third quartiles; *whiskers* most extreme data points not considered outliers, + outliers).
(TIF)
