# Supplementary figures and images for: Automatic labeling of molecular biomarkers of immunohistochemistry images using fully convolutional networks
Source: PLoS One. 2018 Jan 19;13(1):e0190783. doi: 10.1371/journal.pone.0190783 (PMC5774709; doi:10.1371/journal.pone.0190783)

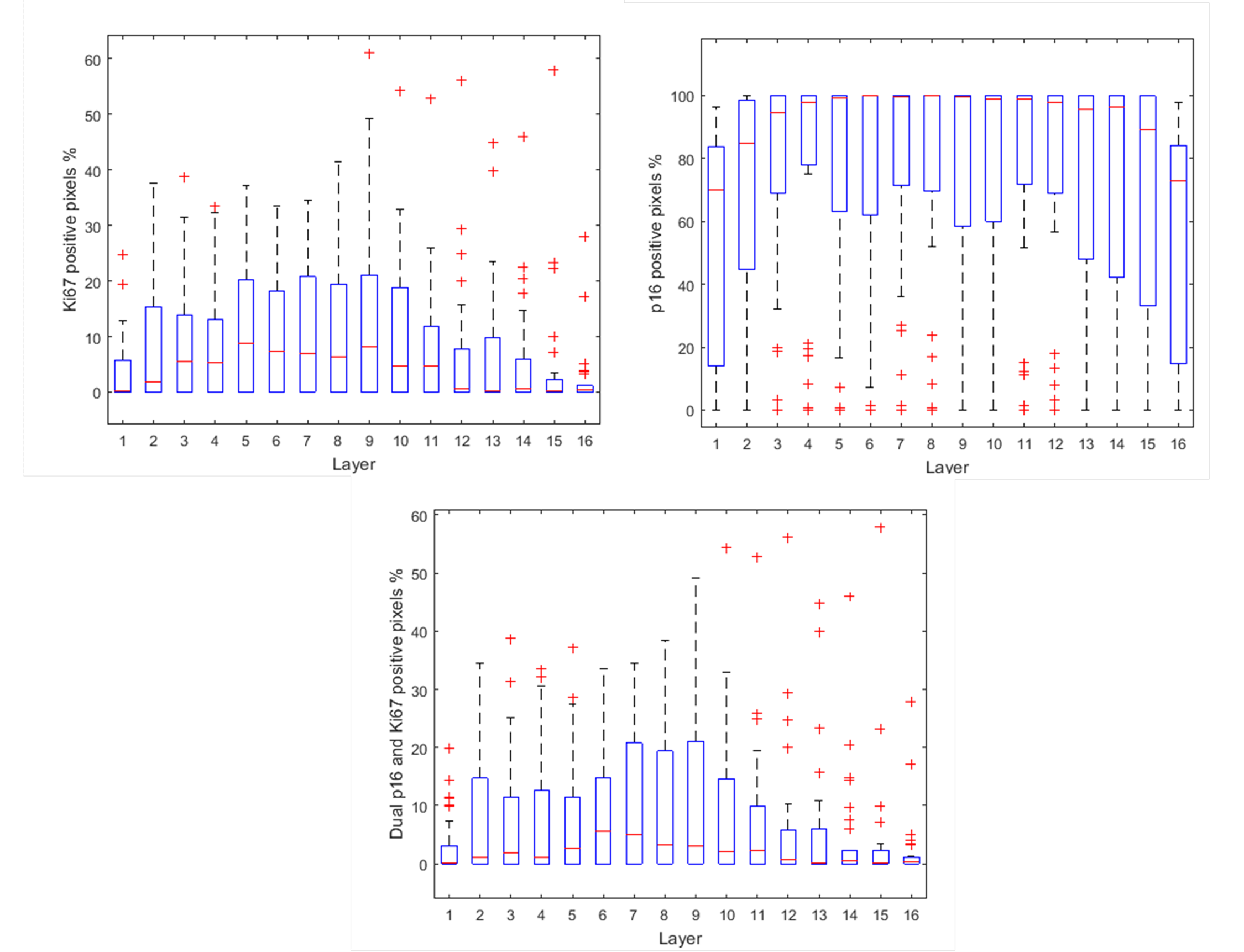

Supplement: S1 Fig — Expression of p16 and Ki-67 per layers in CIN3 lesions. The box plot of the percentage of pixels classified as Ki-67-positive (top left), p16-positive (top right), and both Ki-67 and p16-positive (bottom) in the different layers of CIN3 lesions. Layer 1 corresponds to the first layer (i.e. basal layer) and layer 16 corresponds to the most superficial layer. (Central point median; box first and third quartiles; whiskers most extreme data points not considered outliers, + outliers). (TIF) [file pone.0190783.s001.TIF]

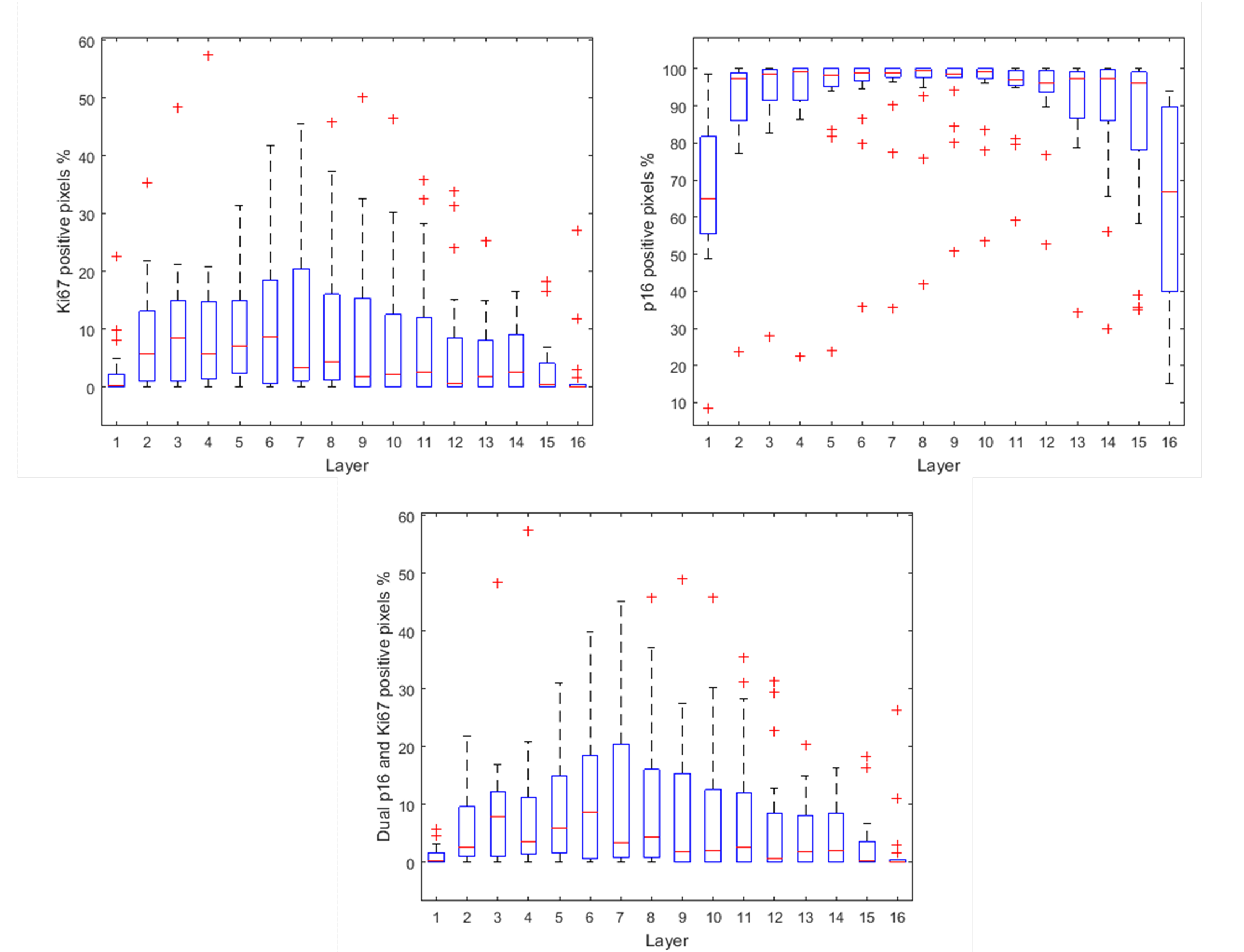

Supplement: S2 Fig — Expression of p16 and Ki-67 per layers in CIN2 lesions. The box plot of the percentage of pixels classified as Ki-67-positive (top left), p16-positive (top right), and both Ki-67 and p16-positive (bottom) in the different layers of CIN2 lesions. Layer 1 corresponds to the first layer (i.e. basal layer) and layer 16 corresponds to the most superficial layer. (Central point median; box first and third quartiles; whiskers most extreme data points not considered outliers, + outliers). (TIF) [file pone.0190783.s002.TIF]

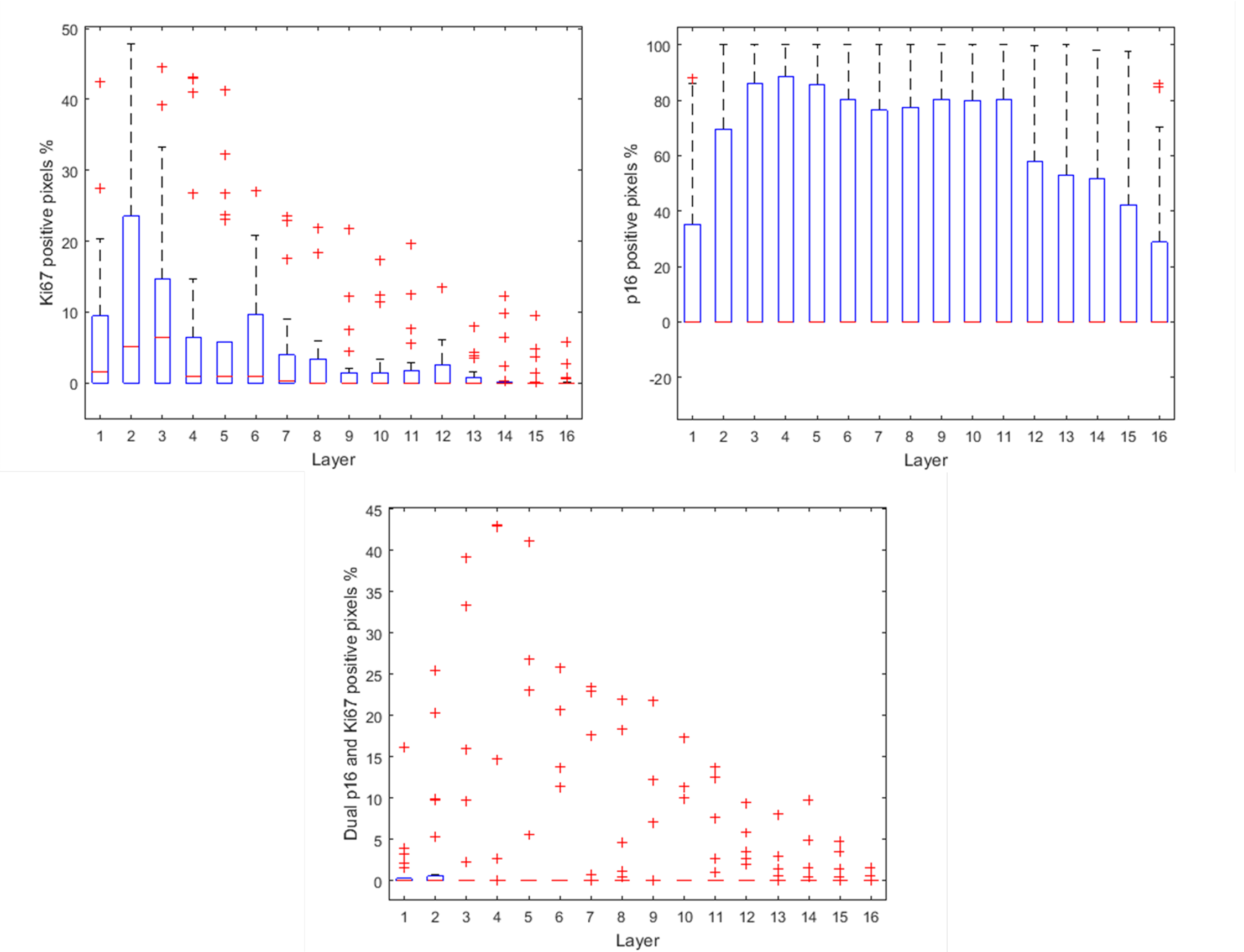

Supplement: S3 Fig — Expression of p16 and Ki-67 per layers in CIN1 lesions. The box plot of the percentage of pixels classified as Ki-67-positive (top left), p16-positive (top right), and both Ki-67 and p16-positive (bottom) in the different layers of CIN1 lesions. Layer 1 corresponds to the first layer (i.e. basal layer) and layer 16 corresponds to the most superficial layer. (Central point median; box first and third quartiles; whiskers most extreme data points not considered outliers, + outliers). (TIF) [file pone.0190783.s003.TIF]

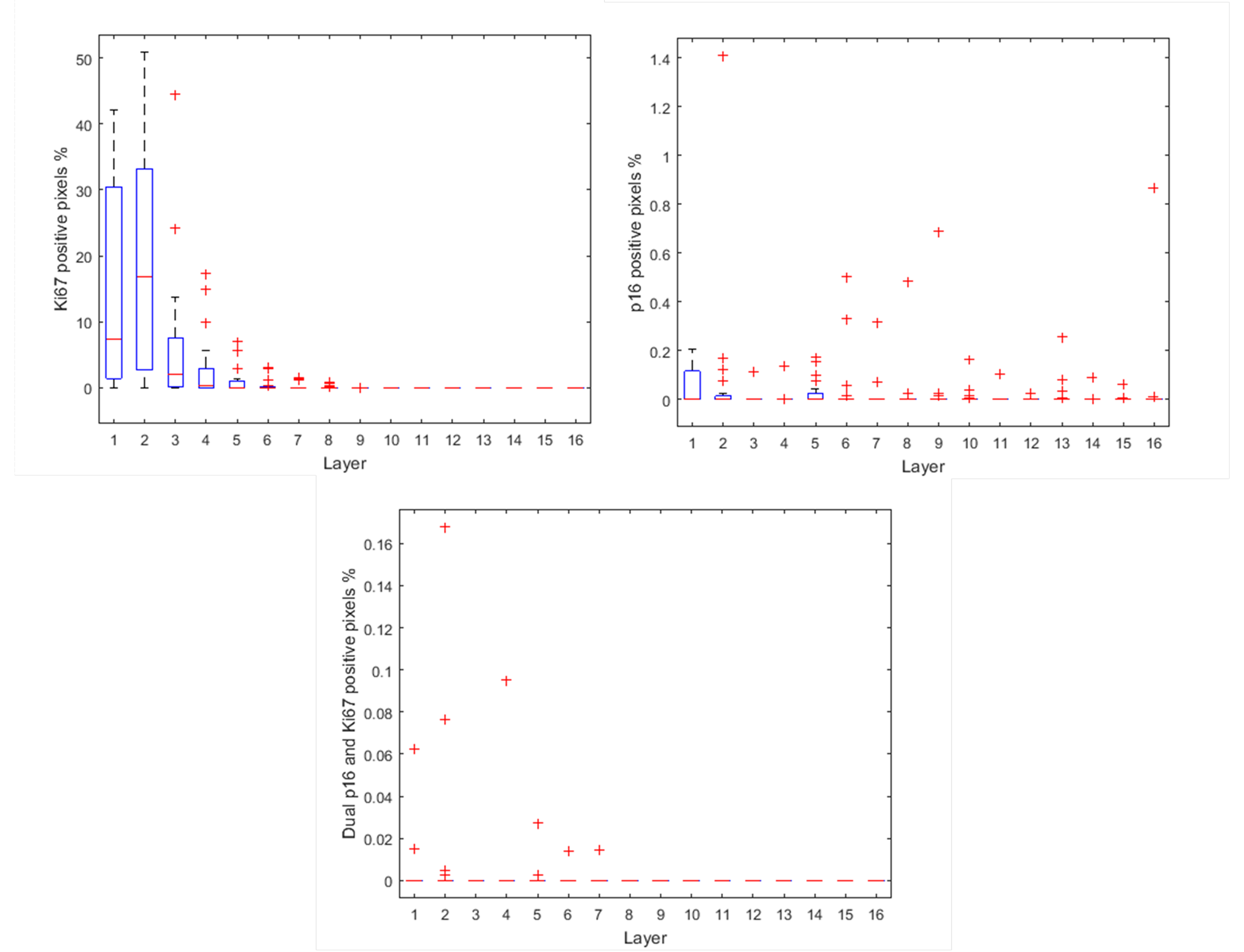

Supplement: S4 Fig — Expression of p16 and Ki-67 per layers in normal squamous cervical epithelia. The box plot of the percentage of pixels classified as Ki-67-positive (top left), p16-positive (top right), and both Ki-67 and p16-positive (bottom) in the different layers of normal squamous cervical epithelia. Layer 1 corresponds to the first layer (i.e. basal layer) and layer 16 corresponds to the most superficial layer. (Central point median; box first and third quartiles; whiskers most extreme data points not considered outliers, + outliers). (TIF) [file pone.0190783.s004.TIF]
